# Label-free diagnostic procedure for hirschsprung’s disease to detect intestinal mucosal characteristics of aganglionosis by Raman spectroscopy with optimized decision algorithms

**DOI:** 10.1007/s10103-025-04579-5

**Published:** 2025-08-29

**Authors:** Yusuke Oshima, Yuki Matsumoto, Katsuhiro Ogawa, Kai Tamura, Rena Yagi, Noritaka Fujisawa, Takashi Katagiri, Shun Onishi, Hidefumi Shiroshita, Tsuyoshi Etho, Tsutomu Daa, Satoshi Ieiri, Masafumi Inomata

**Affiliations:** 1 Faculty of Engineering, University of Toyama, Toyama, Japan; 2 Research Center for Pre-Disease Science, University of Toyama, Toyama, Japan; 3 Department of Gastroenterological and Pediatric Surgery, Faculty of Medicine, Oita University, Yufu, Japan; 4 Graduate School of Science and Engineering, University of Toyama, Toyama, Japan; 5 Graduate School of Pharma-Medical Sciences, University of Toyama, Toyama, Japan; 6 Department of Intellectual Information Engineering, School of Engineering, Toyama, Japan; 7 Department of Pediatric Surgery, Medical and Dental Area, Research and Education Assembly, Research Field in Medical and Health Sciences, Kagoshima University, Kagoshima, Japan; 8 Department of Advanced Medical Personnel nurturing, Faculty of Medicine, Oita University, Yufu, Japan; 9 Research Center for Global and Local Infectious Diseases, Oita University, Yufu, Japan; 10 Department of Diagnostic Pathology, Faculty of Medicine, Oita University, Yufu, Japan

**Keywords:** Keywords: hirschsprung’s disease, Raman spectroscopy, Multiphoton microscopy, PCA, 1D-CNN, LightGBM

## Abstract

**Purpose:**

Hirschsprung’s disease (HSCR) is an intestinal disorder characterized by the absence of nerve cells in parts of the intestinal tract. The definitive diagnosis is confirmed by a full-thickness rectal biopsy to verify the absence of ganglion cells. However, incomplete removal often causes post-operative complications. To establish an optical biopsy technique for targeting mucosa with aganglionosis of HSCR and to confirm its capability by another optical imaging modality and histopathology.

**Methods:**

Raman spectroscopy (RS) is an emerging technique in tissue diagnosis without staining that makes it possible to support conventional diagnostics and therapeutics for achieving more precise outcomes in HSCR. We demonstrate the proof-of-concept for label-free detection of the aganglionic segment in HSCR based on an RS technique in combination with fine-tuned machine learning algorithms.

**Results:**

RS distinguished the characteristics of aganglionic segments in the mucosal surface of the lesion. The altered morphology was confirmed by multiphoton microscopy. In addition, discrimination models were built and evaluated by convolutional neural networks and the decision tree combined with gradient boosting framework.

**Conclusion:**

The proposed method and model show a high accuracy above 90% and a pseudo-blind examination involving three HSCR patients implies the feasibility for clinical application. (195 words)

**Supplementary Information:**

The online version contains supplementary material available at 10.1007/s10103-025-04579-5.

## Introduction

Hirschsprung’s disease (HSCR) is a congenital motility disorder in the intestinal tract. HSCR is characterized by the absence of the enteric nervous system (ENS) in a segment of the distal colon [1–3]. The estimated prevalence of HSCR is 1: 5000 live births. Males are more impacted than females at a ratio of approximately 4:1 [4, 5]. HSCR patients show chronic constipation and abdominal swelling. They may suffer from severe complications, including acute enteritis or toxic megacolon.

The peripheral nervous units involved in ENS are anatomically known as Auerbach’s (myenteric) plexus and Meissner’s (submucosal) plexus. In the intestinal tract of HSCR patients, ganglion cells are absent in innervation areas (aganglionosis). Colorectal aganglionosis is due to the failure of cell migration and differentiation from neural crest in the intrathecal direction during the fetal stage [2, 6]. Aganglionosis is usually connected with hypertrophy of nerve fibers, which are positive for histochemical staining of acetylcholinesterase (AChE) [3, 7]. HSCR treatment involves surgery to completely remove the part of the intestinal tract lacking ganglion cells. The resection margin can be decided by an intraoperative biopsy.

The diagnostic scheme for HSCR requires both a frozen section to evaluate AChE activity and standard HE staining on a formalin-fixed tissue section. However, the traditional biopsy protocol is invasive. Moreover, an accurate diagnosis depends on numerous factors, including the specimen collection site, representativeness of the slice cutting, and biopsy methods (e.g., transmural, submucosal, and serosal-muscular).

To date, optical biopsy techniques based on multiphoton microscopy (MPM) and Raman spectroscopy (RS) have been reported as a non-invasive diagnosis for HSCR and to visualize the distribution of ENS in a suspicious segment of the intestinal tract [8–10]. MPM is a powerful tool for real-time observations of intact tissue with a sub-micron resolution and achieves a good penetration depth. Aggarwal et al. demonstrated that MPM imaging detects Auerbach’s plexus ganglia in mice [8]. In principle, MPM can detect autofluorescence (AF) signals from the ganglion cells due to intrinsic fluorophores such as nicotinamide adenine dinucleotide (NADH) and flavin adenine dinucleotide (FAD) by suppressing the background originating from an out-of-focal plane.

Previously, we showed that MPM is useful to identify the extent of aganglionosis in the human intestine without a labeling procedure [10]. However, MPM has economical and technical drawbacks as it requires a large objective lens with a high numerical aperture, laser scanning machinery, and a high-performance femtosecond laser source. Although clinical use of MPM remains challenging, it is applicable to in vivo cell and tissue imaging in small animals or as a complementary tool for histopathological examination of human intact organs [9–13].

RS holds promise as an alternative technique to provide chemical information of molecular vibrations, which can be used to identify and quantify molecules in cells and tissues [9, 10, 14–16]. We previously demonstrated that RS holds potential as a label-free detection method for intraoperative pathological diagnostics [17]. In our previous work on a new diagnostic approach for HSCR based on an RS technique, the Raman spectra of each layer of the gastrointestinal wall, the mucosa, submucosa, muscularis propria, and serosa of the adult human rectal wall were characterized, and label-free detection of the ganglion cells between the inner circular muscle and outer longitudinal muscle was demonstrated on formalin-fixed and paraffin-embedded (FFPE) tissues of HSCR patient by RS analysis [10]. However, the microscopic approach is currently limited by its narrow measurement range as it is suitable only for tissue surfaces (e.g., mucosa or serosa).

In HSCR-associated enterocolitis, morphological and functional alterations in the mucus layer are linked with aganglionosis due to increased goblet cell proliferation and differentiation in the colonic epithelium [2], and intestinal barrier function, immune response, and the microbiome [18]. Although the underlying pathogenesis of HSCR-associated enterocolitis is not fully understood, the dysfunction of colonic mucosal innervation affects mucosal properties. We hypothesize that utilizing advanced optical biopsy methods such as MPM and/or RS technique may detect changes before enterocolitis onset in HSCR patients.

Here, we aim to develop a new optical biopsy technique for targeting mucosa or serosa with aganglionosis of HSCR. We evaluate a custom-designed compact Raman optical biopsy system combined with effective machine learning methods. The learning methods include supervised PCA-DA, deep learning based on convolutional neural networks (CNNs), and the decision tree combined with gradient boosting framework (Light Gradient Boosting Machine: LightGBM). MPM is employed to reveal histological properties and validate the discrimination results of RS. Furthermore, a resection margin assessment in the transition zone evaluates the discrimination model between ganglionic and aganglionic tissue.

## Materials and methods

### Clinical sample preparation

Tissue specimens were collected from three HSCR patients who were treated with laparoscopic-assisted transanal pull-through in Kagoshima University (Table S1). The rectal segments, which included aganglionosis, were immersed into a 10% formalin neutral buffer solution and stored until the RS measurements. Another segment was cut horizontally along the longitudinal direction and subjected to histopathological diagnosis to determine the transition area between healthy (labeled as “normal”) and aganglionosis (labeled as “HSCR”). The fixative solution was removed by rinsing with phosphate-buffered saline 30 min prior to Raman microscopic observations.

This study complied with the requirements of the Declaration of Helsinki and was approved by the Ethical Committee of Oita University Hospital (Approval ID: 1371). Informed consent was obtained from the legal guardians of all patients.

### Raman microscopic instrument and measurement

A laboratory-built Raman microscopy system was constructed for intraoperative use (Fig. S1). To acquire the Raman spectra, surgical specimens were transversely cut and divided into three segments: aganglionosis, transition zone, and normal parts. The excitation laser was focused on the mucosal surface. Each measurement involved three 10-second laser exposures. For each patient, 30–50 measurements were performed for aganglionosis segment “HSCR” and “normal”. The transition zone was subdivided into three parts (“A,” “B” and” C,” in order from the closest of aganglionosis), and 10 spectral measurements were collected for each part (Fig. S2).

### Multiphoton microscopy

Multiphoton microscopic observations were performed using an upright multiphoton confocal microscope (A1R MP+, Nikon, Tokyo, Japan). The observation protocol and instrumental configuration are detailed elsewhere [12, 13]. Briefly, second harmonic generation (SHG) and autofluorescence (AF) emission from a 950-nm excitation laser were obtained separately via dichroic filters, a short pass filter (492 nm; for the SHG channel), and a bandpass filter (629/56 nm for the AF channel). A 25x water immersion objective lens (CFI75 Apo 25xW MP/N.A. 1.10, Nikon) was employed to acquire the images with a field of view of 509 × 509 μm. Tissue segments were observed from the mucosal surface or the cross-section to the deep penetrating plane (around 200 μm in depth). Observations were stored as z-stack image sequences (step size of 1.5 μm in the z-axis).

### Data preprocessing and exploratory data analysis

The Raman spectral dataset was preprocessed on MATLAB (MathWorks; Natick, MA) with a baseline correction by the asymmetric least squares smoothing algorithm and normalization followed by principal component analysis (PCA). The labeled clusters (“normal” and “HSCR”) were subsequently characterized by principal component (PC) scores and PC loadings. Discriminant analysis with the PC score values was performed by the support vector machine (SVM) algorithm.

### Data augmentation for machine learning

To obtain a sufficient number of training datasets for effective and robust discriminant analyses on machine learning algorithms, random Gaussian noise was added to the raw spectra. This expanded the original dataset four-fold.

### Discriminant model building and training for 1D-CNN

The architecture of the 1D-CNN model, including an input layer, four convolutional and pooling layers, and three fully connected layers based on the PyTorch framework [19], was constructed by Python. Hyperparameter optimization was automated using the Optuna [20] library. The spectral dataset was imported into the input layer of the 1D-CNN model followed by the convolutional layers for local feature extraction and dimension reduction.

### Light gradient boosting machine (LightGBM)

LightGBM is a boosting framework model proposed by Microsoft [21]. It introduces gradient-based one-side sampling (GOSS), exclusive feature bundling (EFB), and leaf-wise (best-first) tree growth, and independent feature merging techniques based on a traditional gradient boosting decision tree. GOSS eliminates most samples with small gradients and then uses the remaining samples to calculate the information gain. EFB involves bundling mutually exclusive features to reduce the dimensionality [22]. The tree is constructed using the growth strategy of the leaf-wise algorithm to reduce computations. The LightGBM python library was used. Hyperparameter tuning was performed by Optuna [20].

## Results

### Characterization of the Raman spectra of rectal mucosa in HSCR

We acquired the Raman spectra for the rectal segments obtained from three HSCR patients. Raman spectral data were exhaustively measured on the mucosal surface in the surgically resected specimens to elucidate the functional and morphological characteristics in the mucosal layer with aganglionosis. Then we investigated whether aganglionosis altered the Raman spectral features. First, the mucosal tissues of the segments in the normal and aganglionosis samples, which were histopathologically determined, were measured by the Raman microscopy system. The surgical specimens provided 190 Raman spectral datasets of the mucosa of ganglionic (normal) and aganglionic (HSCR) segments (case 1 normal, 20 spectra; case 1 HSCR, 20 spectra; case 2 normal 20 spectra; case 2 HSCR, 50 spectra; case 3 normal, 40 spectra; case 3 HSCR, 40 spectra).

**Fig. 1:**
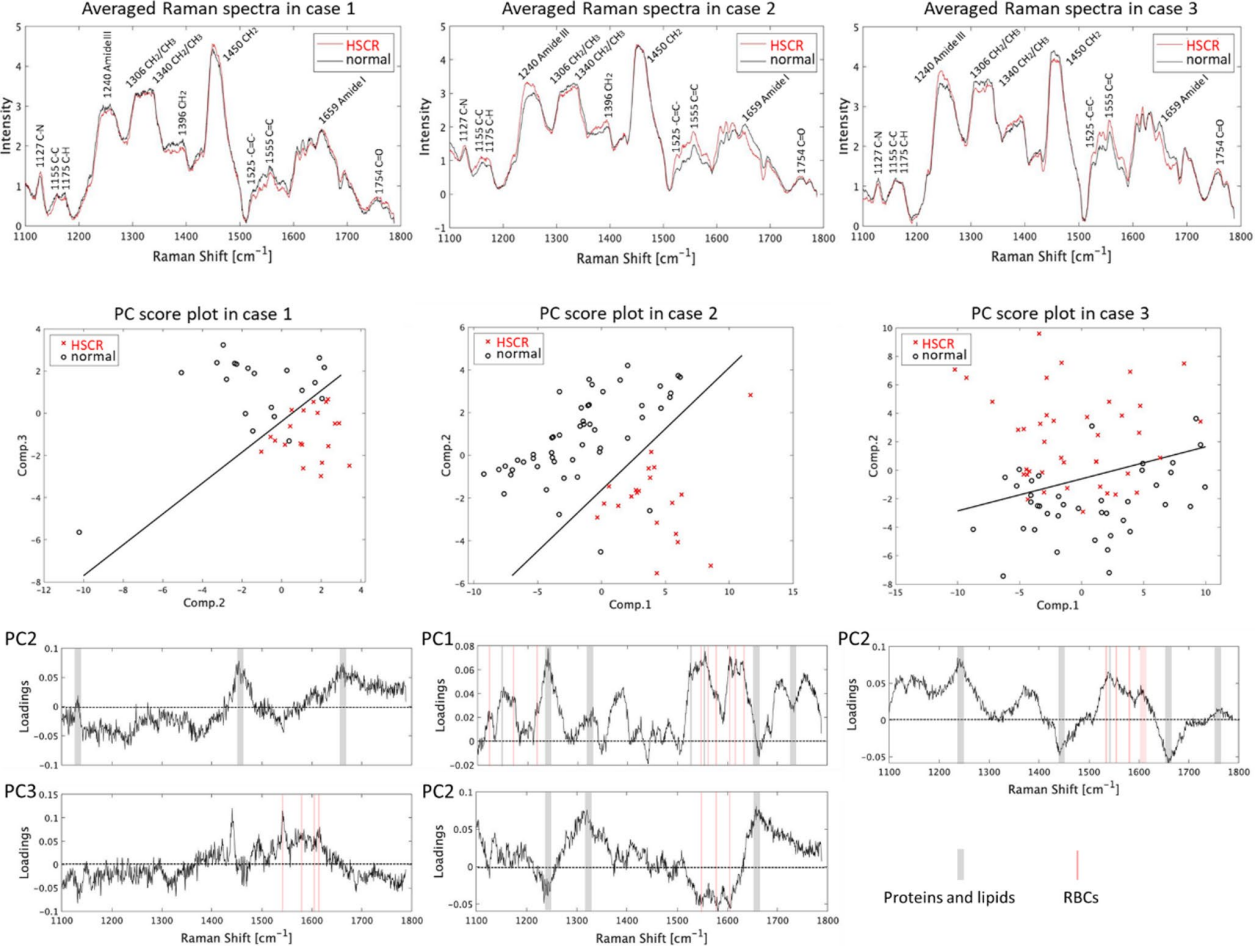
(upper) Comparison of the average Raman spectra measured at the mucosal surface with aganglionosis (HSCR) and the normal portion in the three patients. (middle) Distribution of spectral data plotted two-dimensionally on principal component scores calculated by PCA-DA. (lower) Principal loadings contribute to the discrimination between aganglionosis and normal mucosal tissue. Gray and red lines indicate Raman peaks possibly assigned to typical proteins and lipids of intestinal epithelium, and heme proteins of erythrocytes, respectively

Figure 1 shows the average Raman spectra of aganglionosis and healthy mucosal tissues obtained from the three patients. In the typical spectrum, Raman peaks appeared around 1240 cm^−1^, 1450 cm^−1^, and 1659 cm^−1^, which were assigned to the Amide III band, CH_2_ deformation mode, and Amide I band, respectively. The Raman spectral features slightly differed between the aganglionosis and the normal tissues in each patient.

We then performed PCA to clarify the spectral characteristics in aganglionosis (Fig. 1). The spectral dataset was obviously separated into the clusters labeled as normal and HSCR on the score plots. The discriminant accuracies calculated with SVM were 92.5%, 97.1%, and 78.8% in cases 1, 2, and 3, respectively.

In case 1, the loading spectra of successfully classified segments of normal and aganglionosis, which showed Amide III, CH_2_, and Amide I. The Raman bands, Amide III, CH_2_, and Amide I were attributed to proteins. Additionally, CH_2_ could also be due to the lipid composition in the tissue. Several sharp and small peaks around 1550–1600 cm^−1^ appeared in the loading of PC3. These peaks were possibly assigned to heme proteins in erythrocytes [23]. All averaged Raman spectra included small peaks at 1155 cm^−1^ and around 1525 cm^−1^, which could be derived from carotenoids [24].

In case 2, the small peaks at 1155 cm^−1^ and around 1525 cm^−1^ were positive in the first principal component, indicating that the concentration of carotenoids could be higher in the normal samples compared to that in the lesions. Small and sharp peaks around 1542, 1582, and 1620 cm^−1^ represented Raman peaks of erythrocytes in the blood, and these peaks except that at 1542 cm^−1^ also represented Raman peaks of tyrosine and tryptophan, which could be derived from proteins [24]. Hence, the results confirmed that erythrocytes and proteins were involved in the discrimination between normal and lesion areas. The 1240 and 1659 cm^−1^ peaks were also assigned to amide III, amide I, and protein peaks, indicating that amide III is weaker while amide I is stronger in the lesion area. The 1450 cm^−1^ Raman peak was a lipid peak.

In case 2, the first and second principal components were discriminable with a 97.1% accuracy (Table S2). The Raman peaks contributing to the classification were similar to those in case 1 and were related to carotenoids, erythrocytes, and proteins. However, the wavenumbers of the Raman peaks contributing to the classification differed; the peaks with the highest intensity in the normal area in case 1 were inverted in case 2.

Case 3 had a lower accuracy than those of cases 1 and 2 (Table S2). Regardless, discrimination was possible with an accuracy of 78.8%, which was mainly due to the second principal component. Similar to cases 1 and 2, carotenoids, erythrocytes, and proteins were the main components of the Raman peaks contributing to the classification.

### Morphological observation and quantitative analysis under multiphoton microscopy

**Fig. 2:**
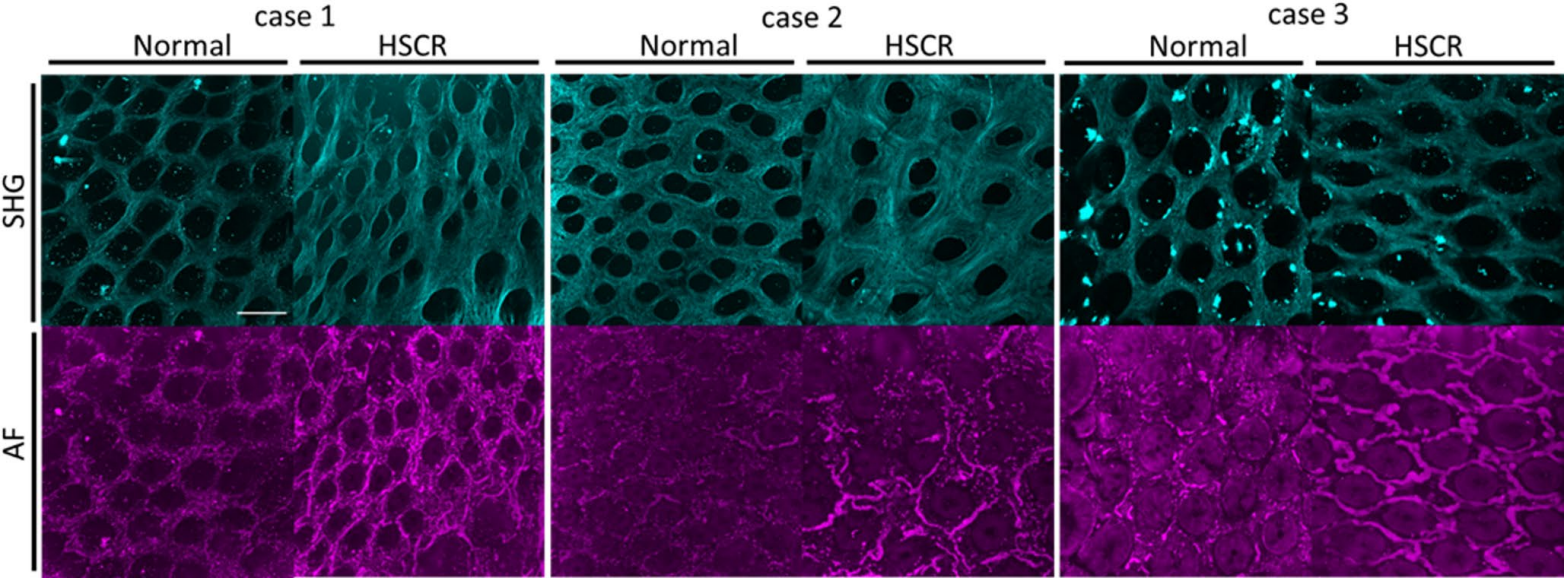
(upper) Second harmonic generation (SHG) images and (lower) autofluorescence images of the mucosal surface with aganglionosis and normal portions. Scale bar indicates 100 μm

Based on the RS observations, we hypothesized that the absence of neurons in the intestine histologically altered the morphology of the mucosal epithelium. Thus, we observed the morphology of the mucosal layer using a two-photon microscope. The samples observed by two-photon microscopy were the same ones used to acquire the Raman spectra. Figure 2 shows the SHG and autofluorescence (AF) images from normal and aganglionosis segments by patient. All images were acquired three-dimensionally. The SHG signals indicated that collagen is distributed in the extracellular matrix.

Two significant differences were observed in the morphology between the aganglionosis and normal portions. First, the lesion area showed a larger proportion of SHG compared to the normal one. Second, AF images at a central wavelength of 629 nm contained an increased number of blood vessel-like structures (Fig. 2), indicating that red blood cells but not blood vessels aggregated in the lesion compared to the normal areas (Fig. S3).

We then quantitatively analyzed the SHG images. The areas of SHG signals per 0.25 mm^2^ in normal and lesion areas were calculated (Fig. S4). In all cases, lesions showed an increased SHG signal area compared to that in normal ones. The findings observed in the intact tissue, accumulation of red blood cells, and altered distribution pattern of the collagen matrix of the mucosal surface could be associated with the Raman spectroscopic measurements with exploratory analysis. In particular, the contribution of Raman peaks from heme proteins derived from erythrocytes provided direct evidence that the Raman spectral features can be distinguished between normal and aganglionic mucosa. Furthermore, the contributions of Amide I, Amide III, and other characteristic Raman peaks, which could be assigned to proteins, were consistent with the morphological changes in the mucosal surface captured by SHG image analysis.

**Fig. 3:**
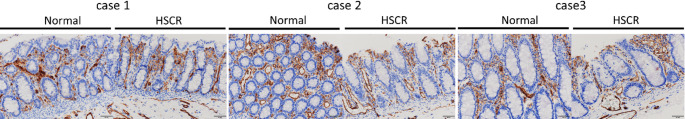
Immunohistochemical (IHC) images of CD31 staining in the mucosal layer at the normal and aganglionic segments. Scale bars indicate 50 μm

Meanwhile, in all cases, we employed immunohistochemical (IHC) staining and Azan staining to confirm the morphological alterations detected by MPM imaging, no remarkable finding in the mucosal tissues at the aganglionic segments was found in the results of histopathological examination in comparison with those at the normal portions. Figure 3 depicts IHC images of CD31 staining identifying vascular structures in the mucosal layer in each case. In case 1, there is no marked difference in diameter or number of blood vessels. In case 2, the vascular distribution is slightly denser in the normal segment. In case 3, slightly more capillary vessels are found in the aganglionic segment. All those findings in histopathological analysis seem to be not remarkable (Fig. 3). In addition, as regards the number and the size of crypts in the mucosal surface, we could not quantify them even in the low-magnification images of IHC and Azan staining (Fig. S5). The distribution of collagen fibers, which was depicted in the SHG images, can be observed in the mucosal surface, but it is difficult to evaluate the morphological characteristics in the histological section.

### Training and testing of discriminant models using a 1D-CNN algorithm and LightGBM

The Raman spectral data successfully captured morphological changes in aganglionosis but the loading spectra contributing to the discrimination results differed among the three cases. In all cases, the number of erythrocytes in the mucosal layer and in the histological morphology of the mucosal layer varied. These differences were related to the discrimination between normal and diseased areas. However, clinical use requires a more robust machine-learning models.

We generated decision algorithms for intraoperative diagnosis of HSCR. Two classification methods were evaluated and compared with the discrimination result of PCA/SVM for each case. The collected Raman spectral data were divided into a training dataset and a testing dataset by case. The classification model was constructed by 1D-CNN or LightGBM. The built models were validated by LOOCV (Leave-of-out cross-validation). In all cases, the classification model by 1D-CNN and PCA/SVM showed almost the same performance, and the use of LightGBM yielded the highest accuracy (Table S2). Data augmentation and preprocessing of the spectral dataset effectively improved the discrimination accuracy (Table S3).

**Fig. 4:**
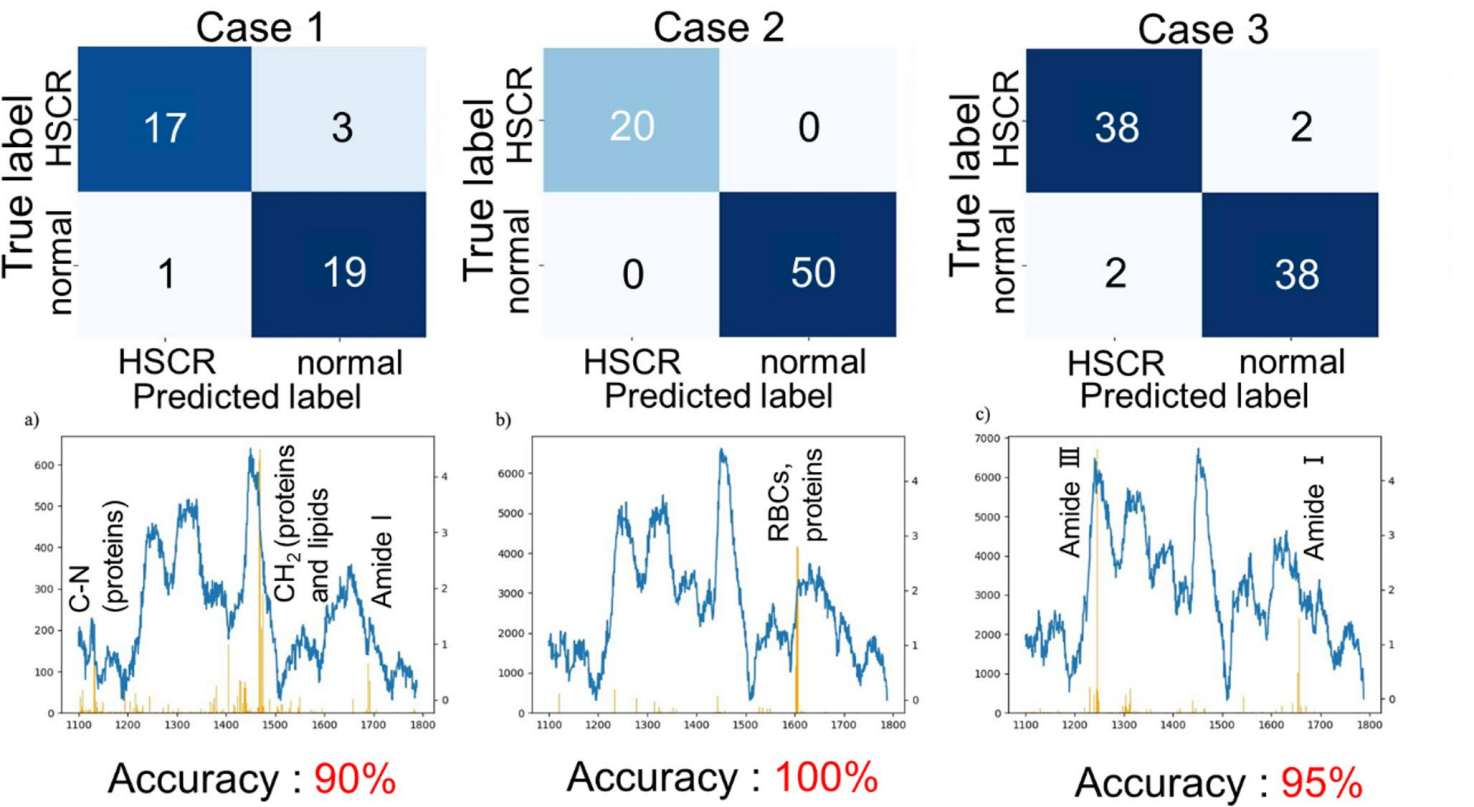
Discrimination results of normal and aganglionosis segments using LightGBM. For each case, (upper) confusion matrix and (lower) representative spectrum (blue lines) and importance value for the discrimination model (yellow lines)

Figure 4 depicts the discrimination results of classification by LightGBM. Plotting the importance, which is an output value of LightGBM, can also be used to examine the wavenumbers used for discrimination. The Raman peak of CH_2_ around 1450 cm^−1^ was mainly used for discrimination in case 1 while the C-N stretching vibration at 1127 cm^−1^, amide I, amide III, and other protein vibrations were partly used. In case 2, the C-N stretching vibration at 1127 cm^−1^, amide I, and amide III were used for discrimination. Additionally, the C = C stretching vibration of erythrocytes or proteins at 1606 cm^−1^ contributed significantly to discrimination. In case 3, amide I and amide III mainly contributed to discrimination.

### Evaluation of the transition zone between aganglionosis and normal tissue

Finally, the transition zone was divided into three groups in the longitudinal direction of the intestinal tract to elucidate the tissue characteristics in the boundary area. The group closest to the lesion was designated as A, the group closest to the normal part as C, and the group in between as Group B. (Note that only group C in case 3 was a normal part in the pathological diagnosis, as shown in Fig. S2). We employed PCA/SVM and 1D-CNN to classify the transition zone.

**Fig. 5:**
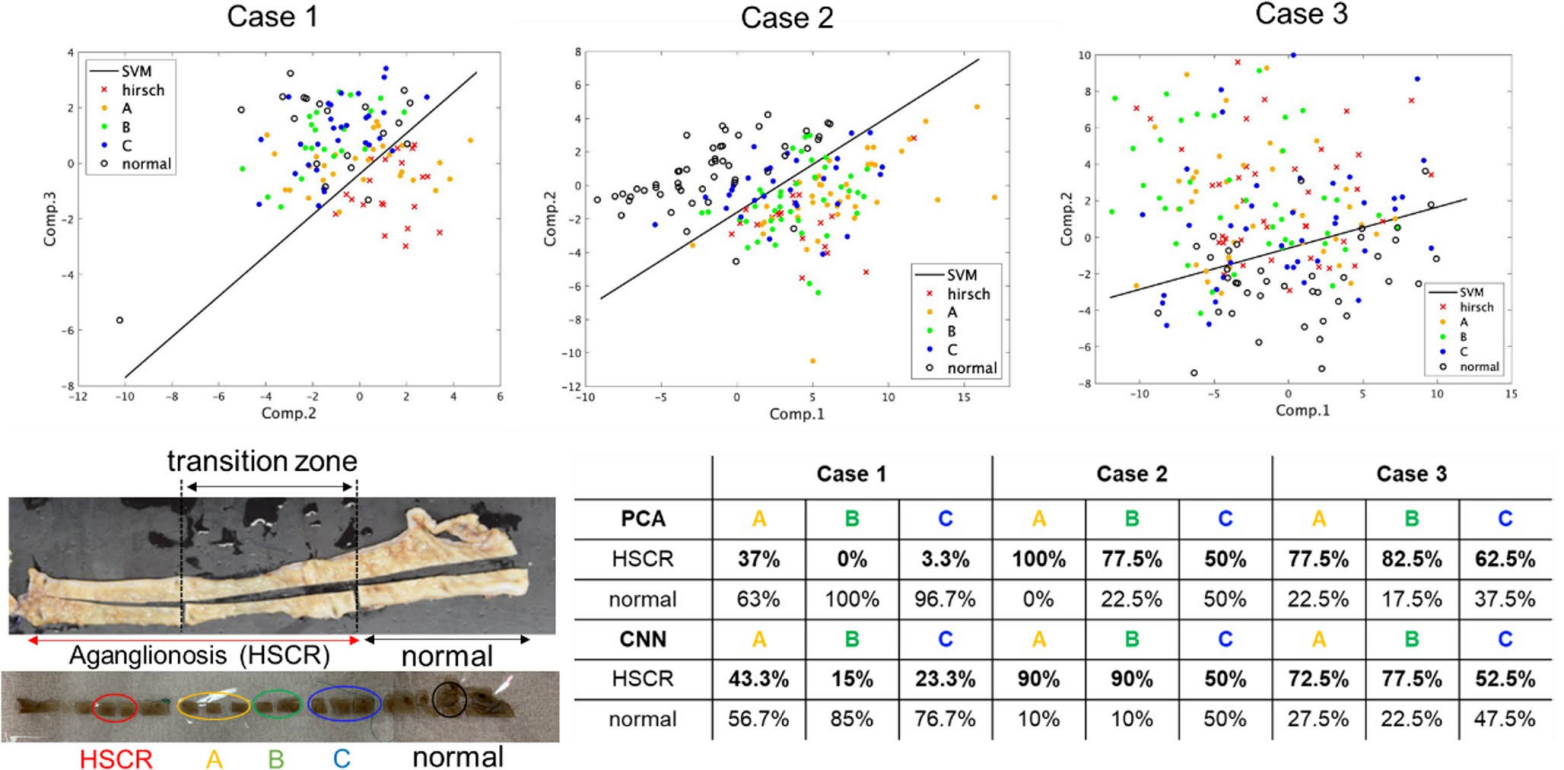
Supervised discrimination models for the transition zone between normal and HSCR constructed by PCA/SVM and 1D-CNN

Since the aganglionic portion could be intermingled in the transition zone, the Raman spectra of the transition zone must be classified as either “normal” or “aganglionosis.” The known (already labeled with normal and HSCR) 190 spectral data were used as the teacher data for classification. Then the Raman spectra of the transition zone were measured and tested (Fig. S2). Figure 5 shows the discrimination results of the Raman spectral data obtained from the transition zone by PCA/SVM and 1D-CNN. The scatterplots indicate the overlay of the classification results of the transition zone onto the discrimination border generated with known spectra data.

In case 1, 37% of spectral data in group A (yellow), which was closest to the lesion, were classified as aganglionosis, whereas most of the spectral data in groups B and C (green and blue) were classified as normal. In case 2, all the spectral data in group A were classified into aganglionosis, while some of the data in groups B and C were discriminated as normal. In case 3, groups A and B were discriminated as aganglionosis, while group C was discriminated as normal. In case 1, the boundary is relatively obvious, and was observed between A and B. In case 2, the transition zone showed that the percentage classified as aganglionosis tended to gradually decrease from group A to C. In case 3, the pathology showed that group C was a normal area, but the aganglionic tract might be longer. The discrimination analysis by 1D-CNN also used the measurement data of normal and aganglionosis areas as the teacher data, and the transition zone was classified in five tests. The results were similar to those of PCA/SVM.

## Discussion

The histopathological characteristic of HSCR is the absence of ganglion cells at the intestinal tract layer of the submucosa and muscularis propria. Currently, segments are determined via an intraoperative biopsy. Although rapid and precise identification of the transition zone during HSCR surgery is crucial to minimize postoperative complications, a practical method to identify the aganglionosis segment rapidly and precisely during surgery does not exist. Improvement of histopathological procedures (e.g., quantification of enteric ganglion density) is one possible solution [25, 26]. Hence, an optical biopsy as a minimally invasive method suitable for endoscopic and laparoscopic surgeries is needed for both surgeons and patients.

We hypothesized that dysfunction of bowel movements caused by aganglionosis might affect the physiological and morphological properties of the entire intestinal tract of the lesion. Herein we demonstrated that an RS technique with PCA successfully identified alterations of the molecular composition in the mucosal epithelium with aganglionosis. Further analyses using MPM revealed that the molecular compositional changes may be due to differences in the size or the number of crypts per unit area in the surface of mucosal epithelium. Moreover, the density of the collagen fiber structure or the accumulation of erythrocytes in the mucosal layer may contribute to the observed difference.

These findings were not captured by the conventional histopathological examination. The optical imaging technique with MPM provided the tissue morphology. In particular, the SHG image was specific for collagen molecules without destructive sample processing (e.g. paraffine embedding, slice cutting, and staining). Our preclinical results indicated that the aganglionic segment, which was determined by routine histopathological diagnosis, is distinguishable by RS and MPM. Namely, RS and MPM could provide indirect evidence to determine the resection margin for HSCR surgery via intraoperative endoscopy and could strongly support conventional rapid pathological diagnosis.

The morphological alteration in the mucosal surface at the aganglionic segment was the most prominent in case 2, which may be due to the time elapsed until surgery after birth. In addition, case 2 had the highest discrimination accuracy. Although case 1 has Hirschsprung-associated enterocolitis (HAEC), no remarkable findings were detected in the mucosal tissue morphology compared to the other cases.

The literature suggests possible mechanisms and the abnormality of mucosal surface in HAEC as follows. With respect to the size and number of crypts, Thiagrajah et al. reported that aganglionosis may induce altered properties in the mucosal surface of HSCR patients prior to the onset of HAEC via increased goblet cell proliferation and differentiation [2]. Keck et al.. demonstrated that the density of AChE + nerve fibers in mucosal tissue is associated with enterocolitis development by immunohistochemistry and RNA seq analysis [18]. Another characteristic found by MPM, the accumulation of erythrocytes in the aganglionic segment, may be associated with a colonic caliber change, which was seen in the transition zone between the ganglionic and aganglionic portions. In all cases, the microvascular structure was unaffected in the aganglionic segment. Therefore, the speculation supports that if the impaired vascular perfusion was due to shrinkage of the intestinal tract, then the specimens fixed with formalin immediately after resection could be captured by MPM observations of the intact tissues via non-destructive approaches.

Some limitations remain toward practical applications of our methodology. These include biological and clinical evidence, instrumentation for intraoperative use, and the generalization performance of the discrimination model. First, the differences in Raman spectral features between normal and aganglionosis reflect alterations of the molecular composition in the mucosal tissues but the pathogenesis of HSCR and allied disorders of HSCR (AD-HSCR) has yet to be fully understood. AD-HSCR refers to a disease group characterized by symptoms and signs similar to those of HSCR, despite the presence of ganglionic cells in the rectum [27]. Further studies should identify both the origin of the histological abnormality in the mucosa and the physiological mechanisms in bowel movement dysfunction in HSCR and AD-HSCR using animal models and additional clinical cases.

Second, our current diagnostic procedure is microscopically made point-by-point in a suspicious portion around the transition zone. Clinical applications require improved instrumentation and acquisition protocol for Raman spectral data. For example, an endoscopic approach using fiber Raman probes [28–30] and the macroview approach using multi-focusing Raman imaging methods [31] are promising techniques to integrate the RS-based intraoperative diagnostic system for practical use.

Finally, the diagnostic accuracy of this technique strongly depends on the training dataset. In fact, the result implies that the discrimination accuracy showed relatively high values applicable for the diagnosis in each case. However, the discrimination model was only built and validated using training and testing data derived from each patient. Nevertheless, we evaluated the generalized performance of the discrimination model to determine the aganglionic segment in the transition zone as a “pseudo-blind examination” and showed that the predicted values of the rate of “ganglionic” and “aganglionic” gradually increased/decreased at the border of the ganglionic and aganglionic segment. To overcome this performance limitation for model discrimination, an increased training dataset from clinical cases and fine-tuning the model for robustness are necessary. (3969 words)

## Electronic supplementary material

Below is the link to the electronic supplementary material.

Supplementary Material 1

## Data Availability

No datasets were generated or analysed during the current study.
