# Supplementary material for: Label-free diagnostic procedure for hirschsprung’s disease to detect intestinal mucosal characteristics of aganglionosis by Raman spectroscopy with optimized decision algorithms
Source: Lasers Med Sci. 2025 Aug 29;40(1):346. doi: 10.1007/s10103-025-04579-5 (PMC12396979; doi:10.1007/s10103-025-04579-5)
Supplement: Supplementary file 1 — Supplementary Material 1 [file 10103_2025_4579_MOESM1_ESM.pdf]

Supplementary materials for

Label-free diagnostic procedure for Hirschsprung's disease to detect intestinal mucosal characteristics of aganglionosis by Raman spectroscopy with optimized decision algorithms

Yusuke Oshima*, Yuki Matsumoto, Katsuhiro Ogawa *et al.*

*Corresponding author

Email: oshima@eng.u-toyama.ac.jp or y-oshima@oita-u.ac.jp

This file includes:

Supplementary Table S1 to S3

Supplementary Figs. S1 to S5

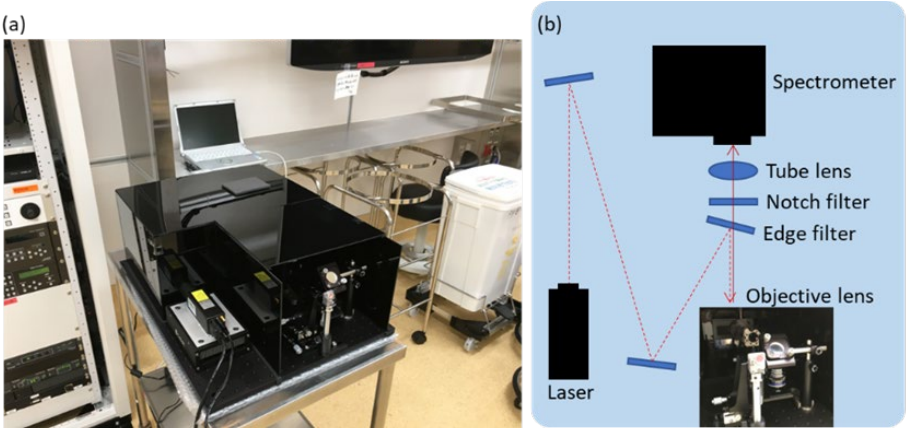

Fig. S1 (a) Custom-built Raman microscopy system and (b) schematic of the optical components and optical light path. A laser source (785 nm, 08-NLD, Cobolt, Solna, Sweden), a spectrometer (M10-TP, 1200/mm, 850 nm blazed, Bunkoukeiki, Tokyo, Japan), an EM-CCD (iXon3-888, ANDOR TECHNOLOGY, Belfast, UK), a 60x water immersion objective lens (UPLSAPO60XW/N.A 1.20, Evident Olympus, Tokyo, Japan), a sample stage, and all optical components were included in one compact box.

Table S1. Patient information

	sex	gestational age at birth	b.w. at birth (g)	age at surgery	b.w. at surgery (kg)	HAEC[a]
case 1	M	40W6D	3008	125D	4.87	P
case 2	M	unknown	2970	1466D	13	N
case 3	M	38W5D	3781	147D	7.9	N

[a]Hirschsprung associated enterocolitis.

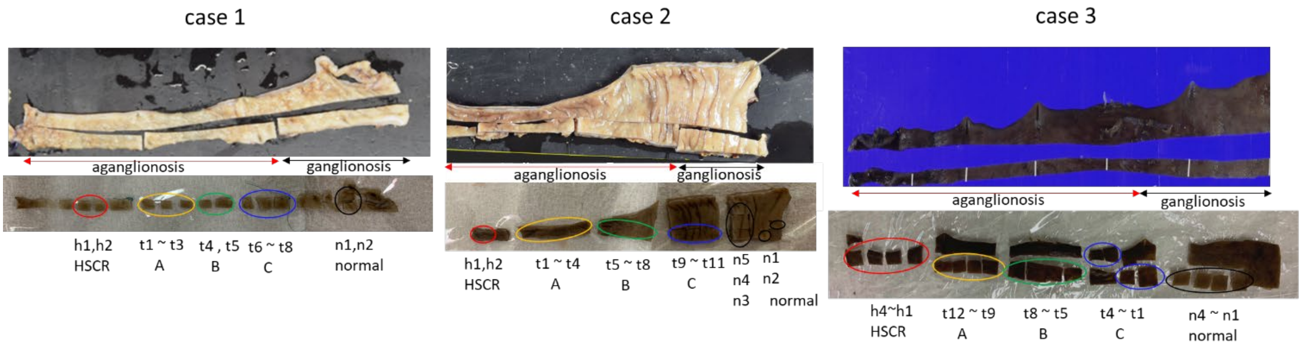

Figure S2. Photographs of the resected specimens. The aganglionosis segment "HSCR" and "normal" one in each patient were provided for the exploratory analysis, the tissue sections in red and black circles, respectively. Regarding the transition zone, the tissue was further divided into three parts "A (yellow)," "B (green)" and" C (blue)," in order from the closest of aganglionosis, and analyzed by decision algorithms.

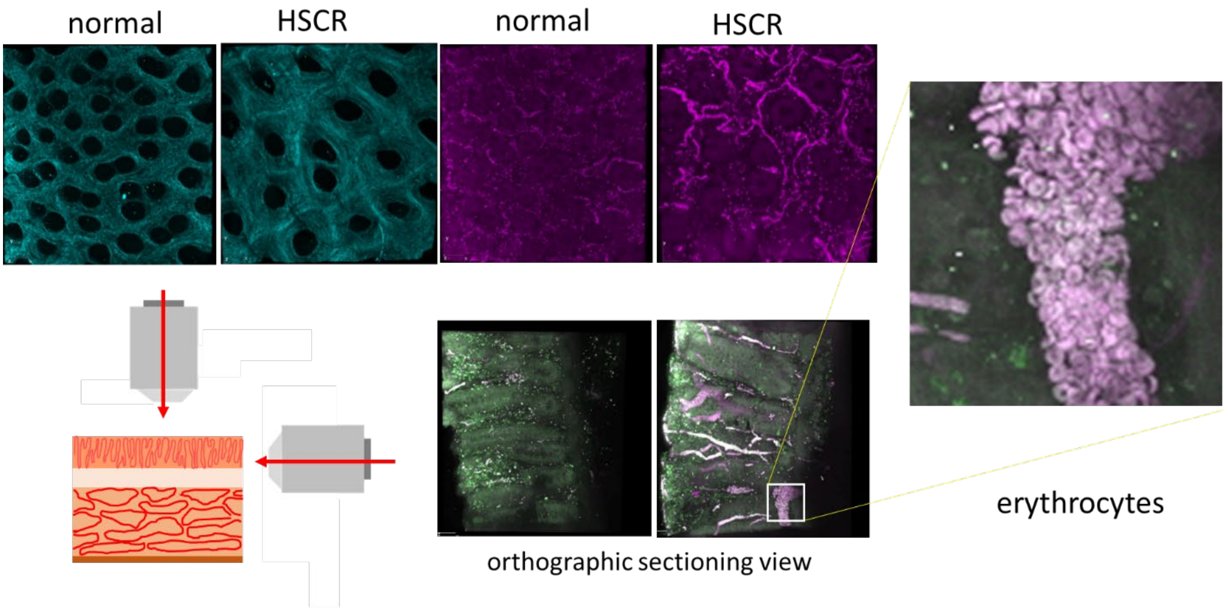

Figure S3. Representative SHG images and AF images were observed in normal and aganglionosis segments in case 2. The blood vessel-like structures observed in the AF image depict the aggregation of red blood cells.

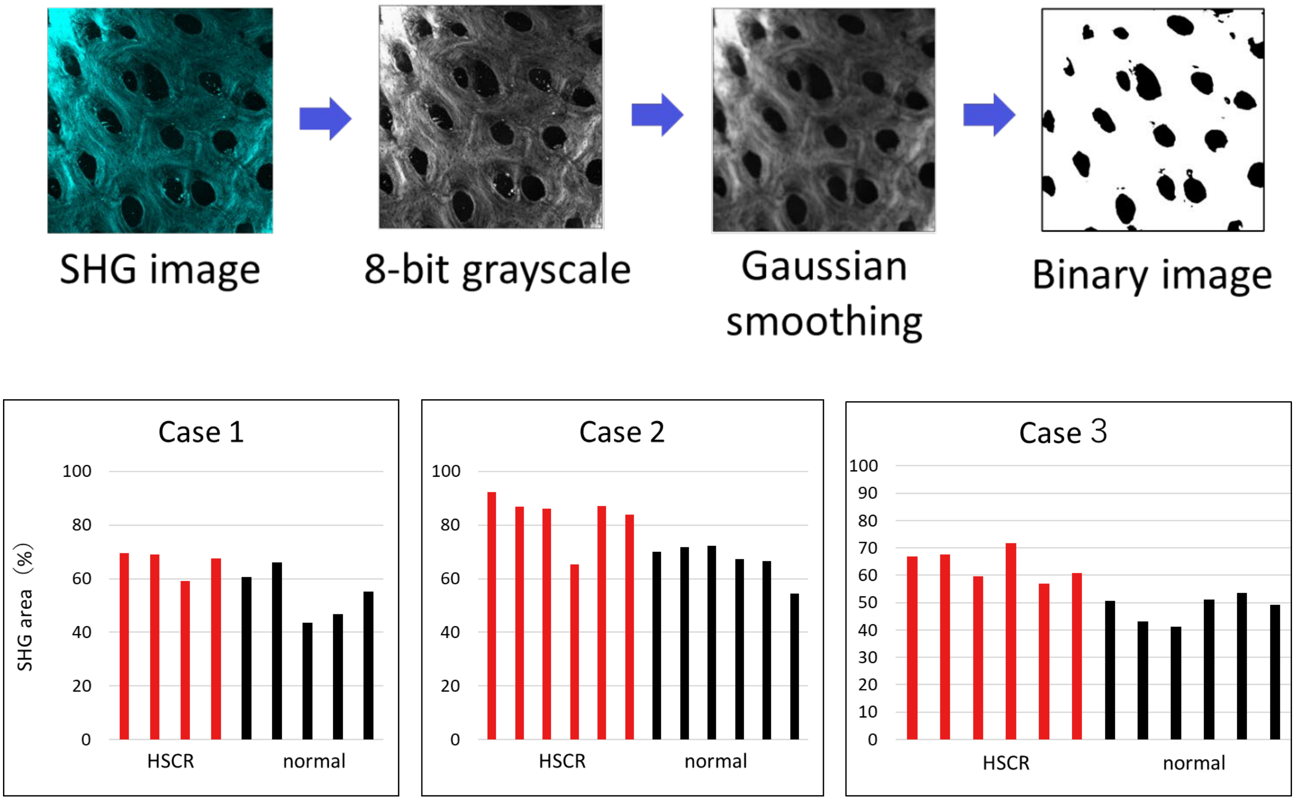

Averaged SHG area (%)	Case 1	Case 2	Case 3
HSCR	66.30	83.62	63.96
Normal	54.48	67.02	48.17
Difference (HSCR-normal)	**+11.82**	**+16.6**	**+15.79**

Figure S4. Quantitative analysis of SHG images. The upper panel shows the image processing flow. Images were converted to 8-bit grayscale images, the noise was removed by Gaussian filter, generating binary images with an appropriate threshold. The middle panel shows the ratio occupied by the SHG signal positive area calculated by the binary images. The values were calculated per 0.25 mm^2 in each region of interest (ROI). The lower panel shows the result of each case. The values of the SHG area in the aganglionosis segment were higher than the normal ones in all cases.

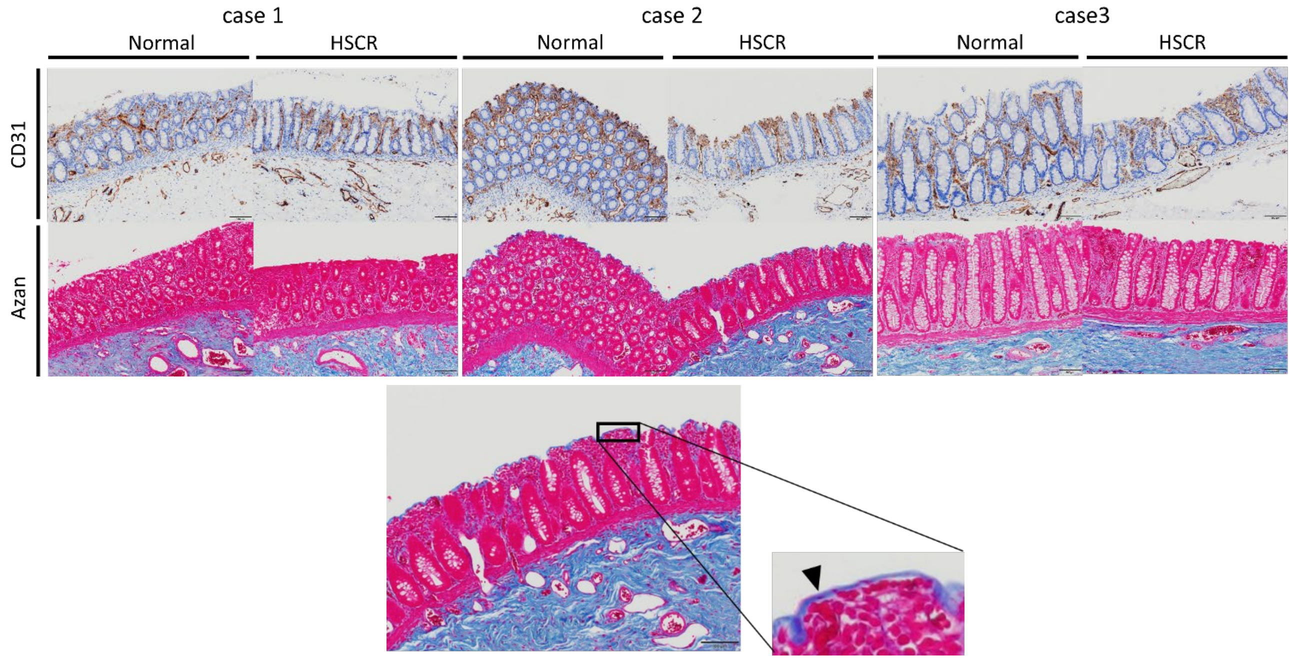

Figure S5. (upper columns) Immunohistochemical (IHC) images of CD31 staining and Azan staining images in the mucosal layer at the normal and aganglionic segments. Collagen fibers are highlighted in blue, and nuclei and erythrocytes are stained red. (lower) A magnified view of the epithelial surface. The layer structure of collagen fibers can be observed in the epithelial surface (arrowhead). Scale bars indicate 100 μm.

Table S2. Discrimination accuracy in PCA/SVM, 1D-CNN, and LightGBM

Discrimination model	Case 1 (%)	Case 2 (%)	Case 3 (%)
PCA/SVM	92.5	97.1	78.8
1D-CNN	80.5	96.6	87.8
LightGBM	90	100	95

Table S3. Comparison of discrimination accuracy in combination with preprocessing of training dataset in 1D-CNN

Preprocessing of training dataset		Case 1 (%)	Case 2 (%)	Case 3 (%)
Augmentation	Baseline correction			
-	-	67.5	86.3	74
✓	-	66.5	90.3	86.3
-	✓	70.5	90	86.8
✓	✓	80.5	96.6	87.8
